# Regulatory role of neuronal guidance proteins in spinal cord injury

**DOI:** 10.4103/NRR.NRR-D-24-00564

**Published:** 2025-05-06

**Authors:** Linyan Tang, Zhi Song, Jie Wang, Shenghua He, Chao Liu

**Affiliations:** 1 Department of Intensive Care Unit, Shenzhen University General Hospital, Shenzhen, Guangdong Province, China; 2 Department of Spine Surgery, Shenzhen Traditional Chinese Medicine Hospital, Shenzhen, Guangdong Province, China

**Keywords:** Eph, ephrin, Netrin-1, neuronal guidance protein, neuronal regeneration, neuronal guidance protein, SEMA3A, SEMA4D, semaphorin, Slit, spinal cord injury

## Abstract

Spinal cord injury is a severe neurological condition with limited neuronal regeneration and functional recovery. Currently, no effective treatments exist to improve spinal cord injury prognosis. Neuronal guidance proteins are a diverse group of molecules that play crucial roles in axon and dendrite growth during nervous system development. Increasing evidence highlights their regulatory functions in spinal cord injury. This review provides a brief overview of the modulation patterns of key neuronal guidance proteins in neuronal axon growth during nervous system formation and subsequently focuses on their roles in neuronal regeneration and functional recovery following spinal cord injury. Neuronal guidance proteins include, but are not limited to, semaphorins and their receptors, plexins; netrins and their receptors, deleted in colorectal cancer and UNC5; Eph receptors and their ligands, ephrins; Slit and its receptor, Robo; repulsive guidance molecules and their receptor, neogenin; Wnt proteins and their receptor, Frizzled; and protocadherins. Localized Netrin-1 at the injury site inhibits motor axon regeneration after adult spinal cord injury while promoting oligodendrocyte growth. Slit2 enhances synapse formation in the injured spinal cord of rats. EphA7 regulates acute apoptosis in the early pathophysiological stages of spinal cord injury, while ephrinA1 plays a role in the nervous system’s injury response, with its reduced expression leading to impaired motor function in rats. EphA3 is upregulated following spinal cord injury, promoting an inhibitory environment for axonal regeneration. After spinal cord injury, bidirectional activation of ephrinB2 and EphB2 in astrocytes and fibroblasts results in the formation of a dense astrocyte-meningeal fibroblast scar. EphB1/ephrinB1 signaling mediates pain processing in spinal cord injury by regulating calpain-1 and caspase-3 in neurons. EphB3 expression increases in white matter after spinal cord injury, further inhibiting axon regeneration. Sema3A, expressed by neurons and fibroblasts in the scar surrounding the injury, inhibits motor neuron and sensory nerve growth after spinal cord injury. Sema4D suppresses neuronal axon myelination and axon regeneration, while its inhibition significantly enhances axon regeneration and motor recovery. Sema7A is involved in glial scar formation and may influence serotonin channel remodeling, thereby affecting motor coordination. Given these findings, the local or systemic application of neuronal guidance proteins represents a promising avenue for spinal cord injury treatment.

## Introduction

Spinal cord injury (SCI) is a devastating neurological event that frequently leads to severe disability and even death, while also imposing significant financial and logistical burdens on healthcare systems. Despite ongoing research efforts, neuronal regeneration and functional recovery following SCI remain highly limited. Clinicians and patients alike are urgently awaiting breakthroughs that could lead to effective treatments and improved prognoses for individuals affected by SCI.

**Figure 1:**
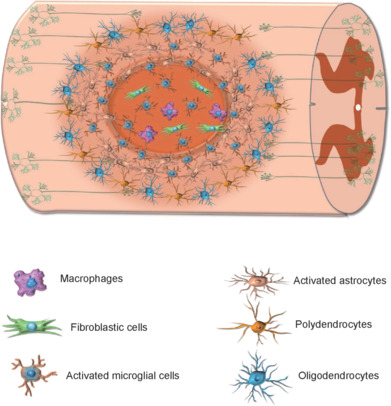
The distribution of different types of cells in lesions after spinal cord injury. Macrophages, fibroblastic cells, and activated microglial cells distribute in the central region of local lesions. Around the central region, activated microglial cells, intermingled astrocytes, and filament proteins restrict the inflammatory reaction area. Polydendrocytes and oligodendrocytes also distribute in the peripheral regions.

SCI manifests with a range of symptoms, including neurogenic shock, paralysis, sensory abnormalities, chronic pain, spasticity, and dysfunction of the cardiovascular, gastrointestinal, urinary, and reproductive systems, along with severe autonomic impairment (Quadri et al., 2020). The pathophysiological changes following SCI are well established and occur in two distinct stages: the primary and secondary phases (Quadri et al., 2020). The primary phase results from an external force that disrupts spinal stability, leading to contusion, laceration, or transection of the spinal cord, either directly or indirectly due to displaced bone or disc fragments (Dumont et al., 2001). Vascular injury in the affected area triggers hemorrhage, edema, and subsequent ischemia (Tator and Fehlings, 1991). Within the first hour after SCI, direct mechanical trauma and ischemia cause irreversible neuronal damage and necrosis in the gray matter, while nerve fiber bundles in the white matter may persist for up to 72 hours (Blight and Young, 1989). During the secondary phase, neuronal membrane disruption and ischemia lead to ionic dysregulation and neurotransmitter accumulation, exacerbating tissue damage (Quadri et al., 2020). In the subacute phase, excessive production of reactive oxygen species and reactive nitrogen species, due to intracellular calcium overload and mitochondrial dysfunction, induces lipid peroxidation and damages proteins and nucleic acids, ultimately leading to cell lysis and neuronal loss (Quadri et al., 2020). The inflammatory response plays a crucial role in this phase, involving rapid neutrophil recruitment, activation of resident microglia, monocyte infiltration, and scar formation (Quadri et al., 2020). Following spinal cord hemisection in mice, lesion areas can be categorized into central and peripheral regions based on the types of recruited cells (**Figure 1**). The central region predominantly contains activated microglia, fibroblastic cells, and macrophages, whereas the peripheral region is populated by activated microglia, reactive astrocytes, polydendrocytes, and oligodendrocytes (Wehrle et al., 2005). Astrocyte activation leads to a phenomenon known as reactive gliosis, in which astrocytes upregulate the production of intermediate filament proteins and restructure the intermediate filament system (Pekny et al., 2014). These reactive astrocytes interact with one another to form a glial scar that helps contain the inflammatory response locally. Research suggests that reactive gliosis has a neuroprotective function, as inhibiting astrocytic scar formation results in increased lesion size and greater neuronal loss following central nervous system (CNS) injury (Bush et al., 1999; Faulkner et al., 2004). However, if glial scars persist beyond the subacute phase, they may impede neuronal regeneration. Studies indicate that suppressing reactive gliosis can enhance axonal regeneration and functional recovery after SCI (Goldshmit et al., 2004; Quadri et al., 2020).

Neuronal guidance proteins (NGPs) are a diverse group of molecules that play essential roles in directing axon and dendrite growth during nervous system development (Chen and Cheng, 2009). Some NGPs remain highly expressed in mature spinal axons and undergo significant changes following SCI. Increasing evidence highlights their regulatory functions in SCI (Chambel and Cruz, 2023). This review provides an overview of the modulation patterns of NGPs in neuronal axon growth during nervous system development and subsequently focuses on their roles in neuronal regeneration and functional recovery following SCI.

## Search Strategy

To investigate the general modulation patterns of relevant NGPs, we conducted a literature search in the PubMed database using the keywords “neuronal guidance protein,” “axonal guidance protein,” and “nervous system formation” or “nervous system development.” To explore the roles of specific NGPs in SCI, we used the keywords “spinal cord injury” along with the names of specific NGPs, such as “Netrin-1,” “Slit,” “Eph,” “ephrin,” “semaphorin3A,” and “Sema3A.” Given the limited number of studies in this field, no time restrictions were applied to the literature search.

## Modulation Pattern of Neuronal Guidance Proteins in Nervous System Formation

The human nervous system is a highly complex communication network that coordinates sensory input, processes information, and orchestrates precise motor responses. This intricate system relies on synaptic connections formed between neurons through dendrites and axons. During embryonic nervous system development, axons elongate and navigate through complex environments to establish synapses with target cells, thereby forming essential neural circuits necessary for proper nervous system function. Given that the human CNS, comprising the brain and spinal cord, contains an estimated 86.1 billion neurons (Herculano-Houzel et al., 2016), understanding how each individual axon migrates to its precise destination remains an enduring yet largely unresolved question. Research has shown that axon growth cones, specialized structures at the tips of axons, detect extracellular cues and respond by modulating the intracellular cytoskeleton to extend or retract filopodia and lamellipodia, thereby facilitating directional movement (Dent et al., 2011). NGPs are among the extracellular signaling proteins that regulate this process.

**Figure 2:**
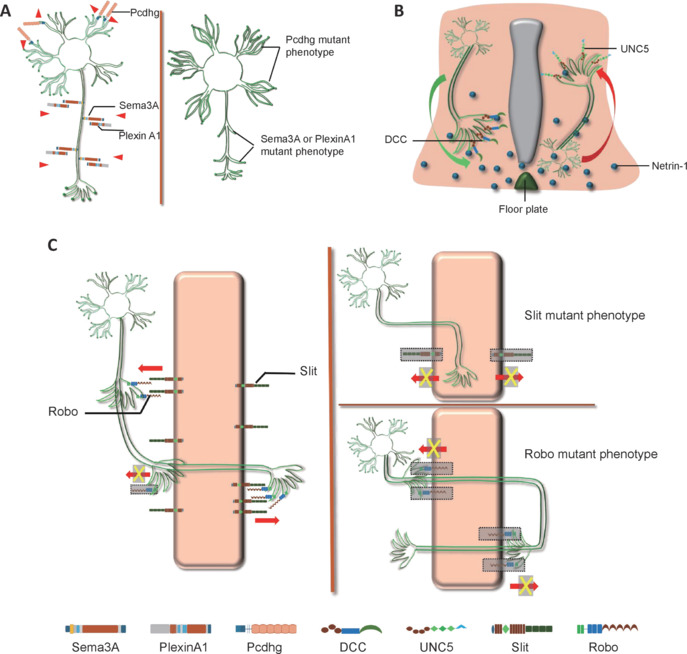
The modulation pattern of NGPs in nervous system formation. (A) Pcdhg provides self-avoidance signals to inhibit the formation of nonfunctional synapses with the same neurons. Pcdhg mutants result in self-crossing dendrites. Sema3A-PlexinA1 interaction acts as a repulsive cue to prevent axon defasciculation and maintain nerve bundling. Sema3A or PlexinA1 mutants result in redundant axon formation and branching. (B) Netrin-1 from the floor plate is attractive to axons of DCC-expressing neurons and repulsive to axons of UNC5-expressing neurons. (C) The midline tissue expresses Slits to prevent axons from crossing it by binding to Robos receptors on axonal growth cones. When the growth cones are ready to cross, Robos are downregulated to silence this repulsion. Once they have crossed the midline, axonal growth cones upregulate the expression of Robos to prevent axon recrossing. In slit mutant Drosophila, axons project to the midline and do not move away from it. Robo mutation results in abnormal axon crossing and recrossing of the CNS midline. CNS: Central nervous system; DCC: colorectal cancer; NGP: neuronal guidance protein.

NGPs are a diverse group of molecules that include, but are not limited to, semaphorins (SEMAs) and their receptors, plexins; netrins and their receptors, deleted in colorectal cancer (DCC) and UNC5; Eph receptors and their ligands, ephrins; Slit and its receptor, Robo; repulsive guidance molecules (RGMs) and their receptor, neogenin; Wnt proteins and their receptor, Frizzled; and protocadherins (Pcdhs) (Leonardo et al., 1997; Kolodkin and Pasterkamp, 2013; Pasterkamp and Kolodkin, 2013; Stoeckli, 2018). These molecules generate attractive or repulsive signals that guide axonal growth either over long distances or through direct cell-cell contact during nervous system development (**Figure 2**).

### Surrounding repulsive signals

Certain NGPs, such as SEMAs and their receptors, plexins (Tran et al., 2007; Pasterkamp and Kolodkin, 2013), act as repulsive cues that prevent inappropriate synaptic connections. For example, Sema3A, the first identified SEMA in vertebrates, was originally named collapsin-1 due to its ability to induce neuronal growth cone collapse (Luo et al., 1993) and inhibit axon branching (Dent et al., 2004). These functions help maintain axon bundling, prevent axon defasciculation, and guide axonal growth and migration through surrounding regions (Dent et al., 2004). During nervous system development, Sema3A is distributed within spinal motor and sensory projections (Tran et al., 2007). In mice lacking Sema3A, spinal nerve projections become disorganized, developing branches outside normal pathways, while the ophthalmic nerve exhibits severe defasciculation during embryogenesis (Taniguchi et al., 1997). This regulatory function relies on its receptor neuropilin (NRP)1 (Yamane et al., 2017) and plexin-A1 but not plexin-Bs (Wang et al., 2018). Binding of Sema3A to plexin-A1 inhibits the activation of the small GTPase Rap1B, thereby restricting the formation of redundant axons (Wang et al., 2018).

### Midline crossing signals

NGPs, including netrin-DCC, netrin-UNC5, and Slit-Robo, play crucial roles in guiding neurons to form commissural axon projections. These projections connect the left and right sides of the nervous system in bilaterally symmetric animals, facilitating essential functions such as integrating bilateral sensory information, coordinating motor commands between hemispheres, and regulating posture and balance. The CNS midline is a critical structure that directs commissural axons to cross once while preventing recrossing. In this process, netrin-DCC functions as a long-range chemoattractive signal, while netrin-UNC5 serves as a chemorepellent signal (Colamarino and Tessier-Lavigne, 1995; Leonardo et al., 1997; Bashaw and Klein, 2010; Pasterkamp and Kolodkin, 2013). During vertebrate spinal cord development, ependymal cells form a transient structure known as the floor plate at the ventral midline (Dickson and Zou, 2010). The floor plate synthesizes netrin-1 (Kennedy et al., 1994; Serafini et al., 1994), which guides commissural axons across the CNS midline by binding to its receptor, DCC, on commissural axons (Keino-Masu et al., 1996). Concurrently, netrin-1 interacts with UNC5, inducing trochlear motor axons to extend away from the floor plate, thereby demonstrating its chemorepellent function (Colamarino and Tessier-Lavigne, 1995; Leonardo et al., 1997). The molecular mechanism underlying netrin-1’s switch from attraction to repulsion has been thoroughly reviewed by Boyer and Gupton (2018). Netrin-1 binding induces DCC homodimerization, forming a signaling platform for attraction. However, when UNC5 is highly expressed on the axonal growth cone membrane, netrin-1 binding leads to heterodimerization between UNC5 and DCC, triggering repulsive signaling through interactions between their intracellular domains (Boyer and Gupton, 2018). Another key chemorepellent system regulating midline axon guidance is Slit-Robo signaling. Midline tissues express Slit, which inhibits axons from either crossing or recrossing the midline through a contact-mediated mechanism (Kidd et al., 1998; Battye et al., 1999). This function depends on Robo receptors located on axonal growth cones. In non-crossing axons, growth cones express high levels of Robo, preventing them from approaching the midline due to Slit-mediated repulsion (Simpson et al., 2000). When axons are prepared to cross, Robo expression is downregulated to silence this repulsion. Once axons have crossed the midline, growth cones upregulate Robo expression to prevent recrossing (Simpson et al., 2000). Mutations in Robo result in abnormal axon crossing and recrossing of the CNS midline (Kidd et al., 1998), while increased Robo2 expression exacerbates commissural defects, preventing axons from crossing altogether (Simpson et al., 2000). In contrast, Drosophila with Slit mutations exhibit axons that fail to move away from the midline due to the loss of repulsion (Simpson et al., 2000).

### Self-avoidance signals

Pcdhs provide self-avoidance signals that prevent nonfunctional synapse formation between the same neurons (Lefebvre et al., 2012; Pasterkamp and Kolodkin, 2013). The Pcdh gene family consists of three closely related gene clusters: α, β, and γ (Phillips et al., 2017), which contain 14, 22, and 22 large exons, respectively (Wu and Maniatis, 1999). These variable exons encode diverse extracellular and transmembrane domains of Pcdhs, while the intracellular domains of Pcdhα and Pcdhγ proteins remain relatively conserved, being encoded by three “constant” exons in their respective clusters. In contrast, the Pcdhβ cluster lacks these constant exons (Wu and Maniatis, 1999). The selective transcription of variable exons, regulated by individual promoters, generates a vast diversity of Pcdh isoforms across different neurons. This diversity is essential for neurons to execute self-avoidance during synapse formation and neural circuit assembly. The importance of this mechanism was demonstrated in an elegant study using Pcdhγ-deficient mice (Chen and Maniatis, 2013). In wild-type mice, retinal starburst amacrine cells extend radial dendrites that avoid contacting their own dendrites while forming synapses with dendrites from neighboring starburst amacrine cells. However, in Pcdhγ-knockout mice, these neurons exhibit abnormal self-crossing dendrites, disrupting the normal self-avoidance mechanism (Lefebvre et al., 2012).

Although various NGPs provide distinct regulatory signals for axon growth, their downstream intracellular effectors are both unique and consistent, primarily involving small GTPases and the cytoskeletal system (Kolodkin and Pasterkamp, 2013; Pasterkamp and Kolodkin, 2013; Stoeckli, 2018). For example, plexin-A1, the receptor for Sema3A, interacts with Rho GTPases such as Rac (Rohm et al., 2000), Rnd1 (Oinuma et al., 2003), and RhoD (Zanata et al., 2002), leading to the stimulation of F-actin depolymerization. Additionally, Sema4D regulates the activation of RhoA or Rac by binding to its receptor plexin-B (Lin et al., 2007). This interaction subsequently influences the formation of actin-myosin filaments and promotes cell motility (Lin et al., 2007). More examples of these signaling pathways will be discussed in later sections.

## Neuronal Guidance Proteins and Neuronal Regeneration in Spinal Cord Injury

The regenerative capacity of neurons and axons in the CNS is dramatically limited compared to the peripheral nervous system (Quadri et al., 2020; Zheng and Tuszynski, 2023; Cao et al., 2024; Franco et al., 2025). This limitation arises from several mechanisms, including the formation of a scar barrier and the presence of environmental inhibitory signals (Quadri et al., 2020; Zheng and Tuszynski, 2023). After SCI, the scar barrier surrounding the lesion consists not only of intermingled astrocytes but also microglia and fibroblasts, which produce a dense extracellular matrix (ECM) containing chondroitin sulfate proteoglycans, fibronectin, collagen, and laminin (Orr and Gensel, 2018; Clifford et al., 2023). These scar barriers form physical obstacles around the lesion site, preventing neuronal and axonal growth and penetration, and thus have been investigated as therapeutic targets for SCI. Relevant research on this topic has been comprehensively reviewed (Orr and Gensel, 2018; Clifford et al., 2023). Additionally, environmental inhibitory signals from glial cells and the ECM play critical roles in negatively influencing neuronal regeneration. These inhibitory factors include Nogo, myelin-associated glycoprotein, oligodendrocyte myelin glycoprotein and their respective receptors and co-receptors; chondroitin sulfate proteoglycans, keratan sulfate proteoglycans, tenascin, and various NGPs (Quadri et al., 2020; Zheng and Tuszynski, 2023). A comprehensive review of other inhibitory factors involved in SCI has also been conducted (Quadri et al., 2020; Zheng and Tuszynski, 2023). This article aims to provide an up-to-date overview of the role of NGPs in SCI.

### Netrin-1 in spinal cord injury

Besides their guidance function in nervous system formation during embryogenesis, some NGPs, such as netrins (Manitt et al., 2001), Slits (Tanno et al., 2005), ephrins (Parrinello et al., 2010) and SEMAs (Pasterkamp and Verhaagen, 2006), have been shown to be expressed in adults and play crucial roles in neuronal regeneration.

Netrin-1 is consistently expressed in rodent spinal cords from the embryonic stage through adulthood (Manitt et al., 2001; Zhu et al., 2025). However, this persistent expression of NGPs is not a universal phenomenon. For instance, Netrin-3 is present in embryonic rat spinal cords but is not expressed in adults (Manitt et al., 2001). In adult rats, Netrin-1 is expressed by various spinal interneurons and motoneurons, including oligodendrocytes (but not astrocytes), interneurons in the dorsal horn, and motoneurons in the ventral horn. As mentioned earlier, Netrin-1 functions as a long-distance signaling molecule for neuronal axon guidance during nervous system development, although it is also present on cell membranes in the embryonic CNS (Serafini et al., 1994). In adult spinal cords, however, the majority of Netrin-1 is membrane-associated and localizes to the outer surface of the cytoplasmic membrane or the ECM (Manitt et al., 2001). Additionally, adult spinal cords express Netrin-1 receptors, including DCC (Rajasekharan et al., 2009), neogenin, UNC5h1, and UNC5h2 (Manitt et al., 2001). The membrane-associated expression of Netrin-1 and its receptors suggests that, in adult spinal cords, Netrin-1 may function through contact-mediated interactions between neurons, oligodendrocytes, and the ECM. This contact-dependent role of Netrin-1 has been further demonstrated to influence neuronal regeneration after SCI.

As mentioned earlier, Netrin-1 functions as a chemoattractant when binding to DCC on neurons but switches to a chemorepellent when interacting with UNC5. Consistent with its dual role in nervous system development, Netrin-1 exhibits both inhibitory and facilitatory effects on neuronal outgrowth and regeneration after SCI, depending on its receptor binding.

Löw et al. (2008) demonstrated that Netrin-1 inhibits motor neuronal regeneration after SCI. Their study revealed that Netrin-1 expression initially decreased during the first week following T7 spinal cord transection in rats but subsequently recovered to levels comparable to those in a normal spinal cord in the following weeks. They also observed that UNC5, a Netrin-1 receptor, was specifically expressed by rubrospinal and corticospinal motor neurons in the gray matter and that its expression decreased following spinal cord transection. In *in vitro* experiments, they found that Netrin-1 inhibits neurite outgrowth and axon regeneration, as neutralizing Netrin-1 significantly enhanced neuronal outgrowth on myelin extracts from adult spinal cords. To confirm this inhibitory function *in vivo*, they induced cervical (C3) SCI in rats and transplanted netrin-1-overexpressing fibroblast grafts into the injury site. Compared to netrin-free fibroblast grafts, the Netrin-1-enriched environment significantly inhibited motor axonal outgrowth and reduced axon density three months after injury. This inhibitory effect was shown to be dependent on the UNC5 receptor on motor axons. Thus, Löw et al. (2008) identified Netrin-1 as an inhibitor of motor axonal growth following adult SCI.

The inhibitory function of Netrin-1 was further demonstrated in a study involving adult neural progenitor cells (NPCs; Petit et al., 2007). NPC migration is highly restricted in the mature CNS, contributing to the limited neuronal regeneration and poor functional recovery of SCI, compared to the peripheral nervous system (Ferretti et al., 2003; Petit et al., 2007). In their study, Petit et al. (2007) cocultured injured spinal cord slices with adult spinal cord progenitor cells and observed that white matter in injured spinal cord slices expressed higher levels of Netrin-1. As a result, spinal cord progenitor cells migrated away from the injured spinal cord, and further analysis confirmed that this repellent cue was specifically due to Netrin-1, rather than Slit-2 or Ephrin-B3.

In contrast to its inhibitory role, other studies have demonstrated that Netrin-1 promotes axon and dendrite growth on oligodendrocytes following SCI (Lu et al., 2023). Lu et al. (2023) explored the function of Netrin-1 in SCI using engineered exosomes derived from netrin-1 modRNA-transfected bone marrow mesenchymal stem cells. Their findings showed that EX-netrin-1 increased neurofilament density in injured spinal cord tissue, suggesting a promotive role in oligodendrocyte regeneration. This facilitatory function may be explained by the fact that oligodendrocytes express DCC, a receptor of Netrin-1 (Rajasekharan et al., 2009). Further supporting this function, Han et al. (2017) conducted a molecular mechanism study in which Netrin-1 overexpression was induced using lentiviral vectors at the rostral and caudal sites of a transected spinal cord lesion. Their results showed that increased Netrin-1 expression enhanced the production of synaptophysin at the rostral site and upregulated growth-associated protein-43 (GAP-43) expression at the caudal site of the lesion (Han et al., 2017). These molecular changes contributed to improved motor function in SCI rats (Han et al., 2017).

The expression of DCC and neogenin in the adult rat spinal cord is significantly reduced compared to their high levels in the embryonic stage (Manitt et al., 2004). In contrast, UNC-5 expression increases in the adult spinal cord relative to the embryonic form (Manitt et al., 2004). Both DCC and UNC-5 expressions are markedly diminished following a spinal lesion (Manitt et al., 2006; Löw et al., 2008). While UNC-5 expression returns to baseline levels within 40 days (Manitt et al., 2006), DCC expression does not recover, even after 7 months (Manitt et al., 2006). These findings suggest that, in the adult spinal cord, UNC-5 predominantly mediates the Netrin-1 signaling pathway, inhibiting neuronal extension, branching, and regeneration following SCI (Manitt et al., 2004).

In contrast to the inhibitory role of local Netrin-1 in neuronal regeneration following SCI, studies involving blood or intraperitoneal injection of Netrin-1 have demonstrated protective effects on acutely injured spinal neurons. First, the serum levels of Netrin-1 in SCI patients were found to be significantly lower than those in healthy controls (Meng et al., 2022). Additionally, Gao et al. (2020) reported that in SCI rats, intraperitoneal injection of Netrin-1 significantly reduced the inflammatory response and apoptosis of injured neurons, as evidenced by decreased levels of nuclear factor kappa-light-chain-enhancer of activated B cells, tumor necrosis factor-α, caspase-9, and caspase-3. These findings suggest that Netrin-1 injection may protect neurons in acute SCI and potentially promote motor neuron recovery. In a separate study, Bai et al. (2017) demonstrated that Netrin-1 enhances autophagy in neurons following SCI in rats. Specifically, intraperitoneal Netrin-1 injection increased the nuclear localization of transcription factor EB in injured spinal neurons and upregulated the expression of lysosomal protease cathepsin D and lysosomal-associated membrane protein 1 (Bai et al., 2017). This intervention restored autophagic flux in injured neurons, which was associated with the subsequent inhibition of neuronal apoptosis and motor neuron loss after SCI (Bai et al., 2017).

In summary, local Netrin-1 at injury sites appears to inhibit motor axonal regeneration following adult SCI (Löw et al., 2008), while promoting oligodendrocyte outgrowth (Lu et al., 2023). However, the administration of Netrin-1 via blood or intraperitoneal injection protects acutely injured spinal neurons by reducing local inflammatory responses (Gao et al., 2020), enhancing neuronal autophagy (Bai et al., 2017), and ultimately inhibiting neuronal apoptosis (Bai et al., 2017; Gao et al., 2020).

### Slit and Robo in spinal cord injury

Slits are evolutionarily conserved proteins, comprising Slit1-Slit3 in most vertebrates (Blockus and Chédotal, 2016). These proteins are synthesized by midline glia and transported to neuronal axons within the CNS (Rothberg et al., 1990). Slits have a molecular weight of 200 kDa and are structurally organized with four 24-amino-acid leucine-rich repeat (LRR) domains, followed by seven to nine epidermal growth factor repeats, an agrin-perlecan-laminin-Slit/laminin-G-like domain, and a C-terminal cysteine-rich module (Blockus and Chédotal, 2016). The receptors for Slits are Robo proteins, named for the phenotype observed in Drosophila mutants, where commissural axons cross and recross the midline of the ventral nerve cord, producing an axonal “ROundaBOut” or “Robo” (Tear et al., 1993; Blockus and Chédotal, 2016). Mammals express four subtypes of Robo proteins (Robo1–4), all of which are single-pass transmembrane proteins (Gonda et al., 2020). The extracellular domains of Robo1-3 are composed of five immunoglobulin (Ig)-like domains (Ig1–5) and three fibronectin type III repeats (FNIII 1–3), while their transmembrane domains contain several conserved cytoplasmic domains (Blockus and Chédotal, 2016; Gonda et al., 2020). In contrast, Robo4 is smaller than Robo1–3 and contains only two Ig domains and two FNIII repeats in its extracellular domain (Huminiecki et al., 2002). The binding of Slit to Robo is mediated by the LRR2 domain of Slit and the first Ig domain (Ig1) of the Robo receptors (Morlot et al., 2007).

As described above, Slits in the axons of midline neurons inhibit axon crossing or recrossing of the midline by binding to Robo receptors on the axonal growth cone (Kidd et al., 1998; Battye et al., 1999). In addition to their role in axonal guidance, the Slit-Robo interaction also regulates the development of several organ systems, including the cardiac vascular system (Mommersteeg et al., 2013), lungs (Anselmo et al., 2003), and kidneys (Grieshammer et al., 2004). The broad regulatory effects of Slits and Robos are attributed to their modulation of small GTPases and the intracellular cytoskeleton, similar to other Netrin-G protein interactions. The binding of Slit to Robo activates Slit-Robo-GTPase-activating proteins, such as Rho GTPase-activating protein (Vilse), which directly binds to the intracellular CC2 motif of Robo (Lundström et al., 2004). This activation of RhoGAPs leads to the hydrolysis of GTP on Rac1 and Cdc42, but not RhoA (Wong et al., 2001; Lundström et al., 2004). The inactivation of Rac1 and Cdc42 inhibits actin polymerization and neurofilament formation, thus contributing to the repellent function of the Slit-Robo interaction. In individual cells, the activation of Rac and Cdc42 promotes the degradation of local Rho (Wang et al., 2003; Zegers and Friedl, 2014), and Rho, in turn, inhibits Rac activation to ensure that different subcellular compartments can perform coordinated, yet independent, motions (Ohta et al., 2006; Zegers and Friedl, 2014). Thus, it is not surprising that the Slit-Robo interaction has also been shown to regulate RhoA activation (Li et al., 2017).

Moreover, the binding of Slit to Robo has been shown to inhibit N-cadherin-mediated adhesion (Rhee et al., 2002; Gonda et al., 2020). Activation of Robo induces hyperphosphorylation of β-catenin by Abelson (Abl), leading to the dissociation of the cadherin–β-catenin complex. This, in turn, disrupts the cadherin–actin linkage, resulting in a reduction of cadherin-mediated adhesion (Rhee et al., 2002).

As previously described, Slits are expressed in the adult spinal cord. In the adult mouse spinal cord, Slit-1, Slit-2, and Slit-3 are expressed by neurons in the grey matter, but not by glial cells (Wehrle et al., 2005). Jacobi et al. (2014) further demonstrated, using neuronal nuclei antigen counterstaining, that Slit-expressing neurons are primarily interneurons and motoneurons. In addition, their *in situ* hybridization experiments revealed that the Slit receptors Robo-1, -2, and -3 are expressed by projection neurons in corticospinal collaterals in mice. Following spinal cord dorsal hemisection, cells at the center of the lesion site express Slit-1 and Slit-3 (Wehrle et al., 2005; Jacobi et al., 2014), but not Slit-2 (Wehrle et al., 2005). These cells were identified by staining as either macrophagic (microglial cells) or fibroblastic cells (Wehrle et al., 2005).

The altered expression of Slit-2 suggests a potential role in SCI. Li et al. (2017) demonstrated that in injured spinal cords of rats, Slit-2 expression was significantly downregulated, while Robo1 and RhoA levels were increased. Silencing Slit-2 with RNAi lentivirus in SCI rats resulted in a further increase in Robo1 and RhoA expression. However, intrathecal injection of Slit-2 appeared to improve locomotor function in SCI rats. Further experiments indicated that application of Slit-2, or silencing Robo1, significantly enhanced the formation of new synapses in the injured spinal cord of rats.

### Eph receptors and ephrins in spinal cord injury

The Eph receptors (erythropoietin-producing human hepatocellular receptors) are members of the larger tyrosine kinase (RTK) receptor family, comprising one of the most extensive subfamilies of RTKs (Egea and Klein, 2007). Their corresponding ligands, called ephrins (Eph receptor-interacting proteins), bind to these receptors. Based on mutual affinity and sequence homology, the Eph family is classified into two subfamilies: EphA and EphB. The EphA-ephrinA subfamily consists of 6 ephrinA ligands (ephrinA1–6) and 10 EphA receptors (EphA1–10), while the EphB-ephrinB subfamily includes 3 ephrinB ligands (ephrinB1–3) and 6 EphB receptors (EphB1–6) (Aoto and Chen, 2007; Klein, 2009). Eph receptors are transmembrane proteins with three domains: an extracellular domain, a transmembrane domain, and an intracellular domain. The extracellular region contains a ligand-binding domain, a cysteine-rich structure, and two fibronectin type III repeats. The intracellular region includes a juxtamembrane area with two tyrosine residues, a classical protein tyrosine kinase domain, a sterile alpha motif domain, and a PDZ-binding motif (Park and Lee, 2015). Ephrin ligands feature a conserved extracellular receptor-binding domain that enables binding to the corresponding Eph receptor. EphrinAs anchor to the cell membrane via a glycosylphosphatidylinositol anchor, while EphrinBs are transmembrane ligands (Park and Lee, 2015).

Typically, EphA binds to ephrinA ligands, and EphB binds to ephrinB ligands, with notable exceptions. For example, EphA4 binds to nearly all ephrins (Qin et al., 2010), and ephrinA5 also binds to the EphB2 receptor (Himanen et al., 2004). A distinctive feature of the Eph family is its ability to induce bidirectional signaling when both the Eph receptor and ephrin ligand are membrane-bound (Egea and Klein, 2007). Eph-ephrin binding activates the classical Eph receptor tyrosine kinase pathway in Eph-expressing cells (‘forward signaling’) and triggers signaling in ephrin-bearing cells (‘reverse signaling’) simultaneously (Egea and Klein, 2007). Unlike other RTKs that utilize Ras-mitogen-activated protein kinase and phosphatidylinositol 3-kinase-protein kinase B pathways, both forward and reverse Eph/ephrin signaling activate Rho GTPases downstream, regulating the intracellular cytoskeleton (Lisabeth et al., 2013).

This contact-mediated bidirectional signaling system plays critical roles in nervous system development, including guiding radial glial cell migration during cortical formation (He et al., 2015), regulating interneuron tangential migration (Zimmer et al., 2011), and contributing to topographic mapping projections in the auditory (Miko et al., 2007), olfactory (Serizawa et al., 2006), and somatosensory systems (Prakash et al., 2000). Eph signaling also aids midline structure establishment and corpus callosum formation (Twigg et al., 2004).

Beyond their role in nervous system development, Eph family proteins are implicated in neural regeneration following SCI (Murai and Pasquale, 2003). Specifically, they activate pathways associated with synaptic plasticity, such as N-methyl-D-aspartate (NMDA) and non-NMDA receptors, and regulate dendritic spines and synaptic plasticity-related proteins, thereby enhancing neural function recovery. Furthermore, Eph family proteins promote the proliferation, differentiation, and migration of endogenous neural stem cells by activating chemotactic factors (Wilkinson, 2001; Nishida and Okabe, 2007).

#### EphA-ephrinA family in spinal cord injury

After SCI, the mRNA levels of EphA3, EphA4, and EphA7 are significantly elevated. Immunohistochemistry revealed that EphA3, EphA4, EphA6, and EphA8 are secreted in the ventral white matter, with EphA7 exhibiting the highest expression levels in both normal and injured spinal cords (Willson et al., 2002). Figueroa et al. (2006) performed polymerase chain reaction to demonstrate a significant rise in EphA7 mRNA levels following SCI. By inhibiting EphA7 expression in rat spinal cord tissue using antisense oligonucleotides, they observed a marked reduction in apoptotic cell density and a significant acceleration in hind limb movement recovery 1 week post-injury. However, this effect was transient, as 2 weeks post-injury, the treated rats showed no significant difference compared to the control group. They thus hypothesized that EphA7 serves as a regulatory factor for acute cell apoptosis following SCI, playing a crucial role in the early stages of SCI pathophysiology.

Arocho et al. (2011) found that in a rat model of SCI, the increase in ephrinA ligands was correlated with a corresponding increase in their homologous EphA receptors. Immunohistochemistry results demonstrated that 7 days after spinal cord contusion, ephrinA1 ligand, along with glial fibrillary acidic protein and neuronal nuclei antigen, was expressed in spinal cord tissue. This expression significantly increased by day 14 and persisted until day 28. Dual labeling revealed that ephrinA1 was expressed in reactive astrocytes and motor neurons. They investigated the baseline mRNA levels of ephrin (A1, A2, A3, and A5) in adult spinal cord tissue and found that only ephrinA1 expression significantly changed after SCI. Behavioral studies further revealed that a decrease in ephrinA1 expression led to poorer motor performance in rats, suggesting that ephrinA1 is involved in the nervous system’s response to injury.

Irizarry-Ramírez et al. (2005) employed the NYU contusion model to examine the expression patterns of EphA3 at both the mRNA and protein levels after SCI. They found that baseline EphA3 expression was low, but EphA3 mRNA levels increased from 2 days post-injury, with this upregulation persisting for 28 days. Furthermore, the forward signaling induced by EphA3 contributed to the formation of an inhibitory environment for axonal regeneration. These results suggest that EphA3 plays a role in the pathophysiology of SCI.

#### EphB-ephrinB family in spinal cord injury

The EphB-ephrinB subfamily plays critical roles in nervous system development, including the regulation of neural crest cell migration, hindbrain segmentation, migration and proliferation of NPCs, hippocampal axon fasciculation, and the regulation of synaptic plasticity in the spinal cord (Jevince et al., 2006; Liu et al., 2009). Increasing evidence underscores their importance in SCI.

In a study examining the relationship between EphB-ephrinB signaling and injuries caused by sciatic nerve damage and chronic constriction injury from dorsal root transection, Song et al. (2008) found that the expression of EphB1 and ephrinB1 in the spinal cord and dorsal root ganglia increased significantly in a time-dependent manner. Bundesen et al. (2003) also demonstrated that after thoracic spinal cord transection, protein levels of ephrinB2 and EphB2 began to rise on the first day post-injury, peaking by day 14. After SCI, ephrinB2 and EphB2 were bidirectionally activated in astrocytes and fibroblasts, ultimately leading to the formation of a complete astrocytic-meningeal fibroblast scar. This resulted in the creation of neuroglial scars and the clearance of meningeal fibroblasts. The increased expression of EphB2 and ephrinB2 after SCI and the subsequent bidirectional signaling contribute to the development of glial scars, which adversely affect axonal regeneration.

Yang et al. (2018) investigated the effects of intrathecal injection of ephrinB1-Fc or ephrinB2-Fc into experimental mice. These injections resulted in significant dose- and time-dependent thermal hyperalgesia and mechanical allodynia, followed by elevated levels of caspase-3 and calpain-1 in the spinal cord. Furthermore, the administration of an EphB1 inhibitor between the L5 and L6 vertebrae in mice prevented chronic constriction injury-induced thermal hyperalgesia and mechanical allodynia. Additionally, cell phenotype analysis revealed that the elevated levels of caspase-3 and calpain-1 in the spinal cord following ephrinB2-Fc treatment were associated with neurons, not astrocytes or microglia. These results indicate that ephrinB1/EphB1 signaling mediates spinal injury pain processing by regulating calpain-1 and caspase-3 in neurons.

In separate rat SCI models, researchers Miranda et al. (1999) and Willson et al. (2003) observed a significant rise in EphB3 mRNA levels in the white matter 7 days after SCI (Miranda et al., 1999; Willson et al., 2003). This increase in EphB3 mRNA expression also extended to the ventral horn gray matter and intermediate zone. Colocalization studies in rats revealed elevated EphB3 expression in white matter astrocytes and gray matter motor neurons. Both studies concluded that the increase in EphB3 suppresses axonal regeneration in SCI (Miranda et al., 1999; Willson et al., 2003).

### Semaphorins in spinal cord injury

The SEMA family consists of various proteins that can be membrane-bound, surface-attached, or secreted (Alto and Terman, 2017). These proteins are classified as SEMAs based on their conserved extracellular sema domain, which consists of approximately 500 amino acids (Alto and Terman, 2017). Based on the basic structure and sequence of amino acids, SEMAs are classified into eight types (No authors listed, 1999). Types 1 and 2 SEMAs are found in invertebrates, while types 3–7 are found in vertebrates, and type 8 SEMAs are present in some DNA viruses (Spriggs, 1999; Pasterkamp and Kolodkin, 2003). SEMA3s are secreted proteins, SEMAs 4–6 are transmembrane proteins, and SEMA7A anchors to the plasma membrane via a glycophosphatidylinositol anchor.

The function of different SEMAs is regulated by their main receptors, plexins and NRPs. Plexins, which have nine members, are classified into four types (plexin-A–D in mammals; Tamagnone et al., 1999; Liu et al., 2025). Plexin-A is activated by SEMAs 5 and 6, and the interactions between plexins and SEMAs are stabilized by the co-receptors NRPs (Worzfeld and Offermanns, 2014). NRPs, which are co-receptors for plexins, consist of two members, NRP-1 and NRP-2. Plexin-B interacts with SEMAs 4 and 5, and plexin-C1 binds to SEMA7A (Worzfeld and Offermanns, 2014). SEMA3A and SEMA4E bind directly to plexin-D1, although several SEMA3s interact with plexin-D1 through NRPs (Worzfeld and Offermanns, 2014).

All SEMAs contain an N-terminal sema domain of approximately 500 residues, which forms a seven-blade β-propeller (Gherardi et al., 2004). The sema domain is closely connected to an adjacent PSI (plexin-SEMA-integrin) cysteine-rich domain. SEMAs generally form homodimers in their structure, but heterodimer formation has not been observed to date (Klostermann et al., 1998; Koppel and Raper, 1998; Love et al., 2003). SEMAs regulate homodimerization through interactions with plexins and NRPs (Janssen et al., 2010; Liu et al., 2010; Nogi et al., 2010). Plexins are large transmembrane proteins that consist of ten extracellular domains, one transmembrane domain, and a cytoplasmic region containing a Rho GTPase-activating protein-binding domain (Kong et al., 2016). The extracellular region includes an N-terminal sema domain, three PSI domains, and six Ig domains (Zhang et al., 2021). The N-terminal sema-PSI domain structure on plexin regulates its interaction with SEMA. Unlike the SEMA dimer domain, no dimerization occurs within the plexin domain, and one SEMA domain binds two monomeric plexins to form a SEMA-plexin complex (Koppel and Raper, 1998; Love et al., 2003; Janssen et al., 2010). The specificity of the SEMA-plexin interaction site is determined by different insertions in the sema domain of SEMAs, leading to unique plexin and SEMA specificity.

NRP-1 and NRP-2 share 44% sequence identity and exhibit conserved structures with domains a1/a2, b1/b2, and c (meprin, A5, and μ-phosphatase) in their extracellular regions (Broz et al., 2022).

The SEMA-plexin signaling system plays vital roles in human physiology and pathology, especially in the nervous and immune systems and in angiogenesis (Neufeld et al., 2012; Pasterkamp, 2012; Gu and Giraudo, 2013; Kumanogoh and Kikutani, 2013). They are crucial in regulating the development of the CNS. Numerous studies have shown their role as repulsive or attractive guiding molecules in SCI, with their functions being dependent on timing or neuronal subtype (Koncina et al., 2007).

#### Sema3A in spinal cord injury

In axon guidance of vertebrates, Sema3s are among the most well-studied members of the SEMA family. The Sema3 subfamily includes seven members (A–G), all of which function as secreted proteins that guide axon growth (Derijck et al., 2010). They exert their repulsive or attractive effects by binding to co-receptors NRP-1 or NRP-2 and plexin-A1 on the surface of the growth cone (Derijck et al., 2010), with the exception of Sema3E, which cooperates with plexin-D1 (Chauvet et al., 2007). Specifically, Sema3A binds to NRP-1 (De Winter et al., 2002), while Sema3B, Sema3C, and Sema3F interact with NRP-2 as co-receptors (Chen et al., 1997; Takahashi et al., 1998; Giger et al., 2000).

In the mature nervous system, Sema3A is primarily expressed in cerebral and spinal motor neurons, but not in glial cells. After SCI, Sema3A, along with Sema3B, Sema3C, Sema3E, and Sema3F, is secreted by meningeal fibroblasts in the central region of the scar (Niclou et al., 2006).

Sema3A acts as an RGM by binding to plexin-A1 and NRP-1. Kim et al. (2023) found that Sema3A is expressed in neural stem cells derived from the rat embryonic spinal cord, and knockdown of Sema3A promoted cell survival, neuronal differentiation of neural stem cells, and synapse connection of transplanted neural stem cells in the damaged spinal cord of rats. In models of complete transactional and confusional SCI in rats, Sema3A is secreted from neural scars around the spinal cord lesion, and spinal descending fibers are unable to pass through these Sema3A-positive scars (De Winter et al., 2002). Similarly, Sema3A expression was strongly induced by fibroblasts in scars following spinal dorsal column injury, and the expression of NRP-1 and plexin-A1 was observed in injured dorsal root ganglion neurons. An exclusion zone was formed by the Sema3A-positive scar where axonal regeneration was blocked (Pasterkamp et al., 2001). Compared to motor neurons, Sema3A secreted by Schwann cells preferentially affects sensory nerves by preventing neurite growth through binding with NRP-1 (Shen et al., 2023). The growth of sensory neurons after SCI is regulated by the Sema3A/NRP-1/plexin-A1/RhoA/ROCK axis, and miR-30b was found to inhibit this pathway, promoting sensory recovery (Wang et al., 2020).

Due to the efficient repulsive effects of Sema3A, several inhibitors of Sema3A have been investigated in SCI models to evaluate their therapeutic potential. For instance, decorin was used to suppress Sema3A expression in neuron scar tissue following CNS traumatic injury in adult rats, and this was shown to promote sensory axon growth (Minor et al., 2011). Inhibition of the Sema3A/NRP-1 signaling pathway in oligodendrocytes enhanced motoneuron survival in the ventral horn of the damaged spinal cord, improving the Basso-Beattie-Bresnahan score, which represents motor function recovery after SCI (Hu et al., 2022). Dimeric Galectin (Gal-1) inhibited Sema3A by binding to the NRP-1/plexin-A4 receptor complex in a glycan-dependent manner, promoting complete axonal regeneration and restoration of locomotor function (Quintá et al., 2014). Xanthofulvin (SM-216289), a Sema3A inhibitor, was shown to facilitate neuronal function recovery by enhancing axon regeneration, myelination, and angiogenesis while reducing apoptosis (Tohda and Kuboyama, 2011). Additionally, epigallocatechin gallate was found to attenuate the Sema3A-related repulsive effects on axons in SCI (Pasterkamp et al., 2006). Electroacupuncture, which has long been used to improve SCI recovery, effectively reduced the accumulation of polymorphonuclear neutrophils by promoting Sema3A degradation, facilitating the recovery of motor neurons after SCI (Hu et al., 2020). The combination of Sema3A and nerve growth factor also promoted synaptic formation and axon regeneration into the denervation area of the spinal cord, leading to nociceptive functional recovery (Tang et al., 2007). Moreover, Sema3A was considered an inhibitory guidance molecule for nerve growth factor-responsive nociceptive afferents and selectively inhibited neuropathic pain and sprouting induced by nerve growth factor in adult rats (Tang et al., 2004).

In summary, Sema3A is expressed by neurons and fibroblasts in scars around SCI lesions, where it inhibits the outgrowth of motor neurons and sensory nerves. Interfering with or inhibiting the Sema3A signaling pathway promotes nerve regeneration after SCI and facilitates recovery of motor and sensory functions.

#### Sema3C in spinal cord injury

Sema3C is another Sema3s member whose function in SCI has been investigated. In embryos, Sema3C is an attractive signal for cortical axonal migration (Bagnard et al., 1998), and distributes predominantly around the cortical midline in a gradient-like pattern (Niquille et al., 2009; Ruediger et al., 2013; Mire et al., 2018). It can be transiently generated by glutamatergic neurons surrounding the corpus callosum (Niquille et al., 2009; Ruediger et al., 2013; Mire et al., 2018). Sema3C and Sema3B compete with Sema3A to bind to NRP-1, prevent Sema3A-induced growth cone collapse, and act as antagonists of Sema3A (Takahashi et al., 1998).

In SCI, Sema3C has been shown to regulate inflammation and neuronal regeneration. Sema3C was not detectable in mature, uninjured spinal cord neurons of mice and rats (Oschipok et al., 2008). However, its mRNA level was significantly upregulated in rubrospinal neurons after axonal injury induced by transection of the left dorsolateral funiculus at the C4 level (Oschipok et al., 2008). Shen et al. (2024) showed that the expression of Sema3C and its receptor NRP-2 increased in parallel after blunt force SCI in mice, peaking at 7 days post injury and decreasing to control levels by 28 days post injury. They confirmed increased Sema3C in macrophages in the central regions of the lesion and demonstrated that Sema3C contributes to microglial polarization to pro-inflammatory and pro-apoptotic states *in vivo* and *in vitro*. Sema3C induced upregulation of inflammatory cytokine release, which subsequently exacerbated neuronal and myelin damage. In addition, Sema3C attenuated angiogenesis of HUVECs *in vitro*. This modulation was shown to involve activation of the RAGE/NF-κB pathway.

#### Sema4D in spinal cord injury

Sema4s are transmembrane proteins that include seven members (Sema4A-G) in humans. The primary receptors for Sema4s are plexin-Bs and NRPs, although no receptors have been identified for Sema4E and Sema4F (Nojima, 2022). Among this family, Sema4D plays a particularly essential role in SCI.

Sema4D, also known as CD100, was initially identified in the immune system and is involved in various physiological processes, including neuronal development, tissue regeneration, and immune responses. In the nervous system, Sema4D inhibits oligodendrocyte differentiation during development, and its deficiency leads to an increased number of oligodendrocytes in the cerebral cortex (Yamaguchi et al., 2012). It also regulates the formation of GABAergic synapses, with silencing of Sema4D resulting in a reduction in inhibitory synapse density (Raissi et al., 2013). Additionally, Sema4D promotes growth cone collapse by binding to plexin-B1 (Ito et al., 2006). In developing mouse spinal cords, Sema4D is highly expressed in the ventral (motor) lamina but declines to undetectable levels after birth (Worzfeld et al., 2004). However, its expression in spinal white matter oligodendrocytes increases postnatally (Worzfeld et al., 2004).

Sema4D has also been shown to play a critical role in SCI. In adult zebrafish, Sema4D expression is upregulated 4 hours post-injury and remains elevated for 3 days following spinal cord transection. The increased Sema4D primarily originates from motoneurons along the central canal. Compared to Sema4D-knockout neurons, Sema4D-positive motoneurons activate more microglia during the acute phase of SCI and contribute to axon regeneration and locomotor recovery (Peng et al., 2017).

Zhang et al. (2014b) found that in spinal transection rat models, Sema4D expression was upregulated and peaked within the first week after SCI. It was expressed in both endothelial cells and oligodendrocytes, promoting angiogenesis while inhibiting neuronal axon myelination *in vitro* (Moreau-Fauvarque et al., 2003; Zhang et al., 2014b). Knockdown of Sema4D using siRNA lentivirus in oligodendrocytes improved locomotor function recovery after SCI in rats (Zhang et al., 2014b). Further studies demonstrated that Sema4D knockdown led to reduced spinal cord edema two to three weeks post-SCI, along with suppression of inflammatory cytokine release, inhibition of angiogenesis-related factors, and upregulation of axon regeneration-associated genes, including S100b, Erbb2, Notch1, Dcx, and Drd2 (Zhang et al., 2014a).

In mammals, neuronal regeneration and functional recovery after SCI are severely limited compared to species like salamanders, which can fully recover even from complete spinal cord transections. Diaz Quiroz et al. (2014) examined microRNA expression after SCI in axolotls and rats, revealing significant differences in miR-125b levels between the two species. MiR-125b was identified as a downstream regulator of Sema4D, and its expression was dramatically lower in axolotls than in rats following SCI. Inhibition of miR-125b in axolotls led to increased Sema4D expression at the injury site, resulting in regeneration defects. Furthermore, overexpression of Sema4D significantly suppressed axonal regeneration in axolotls, while depletion of Sema4D in astrocytes *in vitro* promoted axonal outgrowth in injured neurons.

The inhibitory effects of Sema4D observed in these studies have motivated preclinical investigations into its therapeutic targeting in SCI. Li et al. (2015) developed collagen-binding proteins, CBD-EphA4LBD and CBD-plexin-B1LBD, to neutralize ephrinB3 and Sema4D. When a collagen scaffold with immobilized CBD-EphA4LBD and CBD-plexin-B1LBD was transplanted into SCI rats, axonal regeneration and locomotion recovery were significantly improved.

#### Sema7A in spinal cord injury

SEMA7A is a glycosylphosphatidylinositol-linked membrane SEMA that was originally identified as a red blood cell antigen and named CDw108 (Bobolis et al., 1992; Pasterkamp et al., 2003). Structurally, it consists of a PSI domain, an Ig domain, and a sema domain (Jongbloets et al., 2013). Sema7A binds to plexin-C1 and β1-integrins as receptors, regulating cell proliferation, differentiation, migration, and maturation across various biological systems, including the nervous, immune, and blood systems.

In the embryonic nervous system, Sema7A and plexin-C1 are expressed in the olfactory system, cortex, hippocampus, and meso-diencephalic dopamine neuron (mdDA) system. Interestingly, plexin-C1 is highly expressed on migrating neurons, whereas Sema7A is more abundant in mature neurons (Pasterkamp et al., 2007). In the embryonic spinal cord, both Sema7A and plexin-C1 are widely expressed, particularly in motor neurons (Pasterkamp et al., 2007). In adult spinal cords, Sema7A is detectable in sensory neurons, motor neurons, oligodendrocytes, and throughout the gray matter, while plexin-C1 is expressed in sensory neurons, motor neurons, and ependymal cells of the central canal (Pasterkamp et al., 2007).

A growing body of research highlights the critical role of Sema7A in SCI. Following SCI, Sema7A expression is upregulated at the lesion site but remains unchanged in adjacent areas. This upregulation peaks at 14 days post-injury and persists for 28 days. After neuronal injury, Sema7A accumulates in retractile bulbs and swollen axons, before being phagocytosed and translocated into lysosomal inclusion bodies of macrophages in the lesion core. At 14 days post-injury, reactive astrocytes highly express Sema7A, intermingling with α1 integrin-positive macrophages. This interaction persists until four weeks post-injury, contributing to the formation of the glial scar (Kopp et al., 2010). These findings suggest that Sema7A plays a role in glial scar formation and may influence neuronal regeneration following SCI.

Serotonergic neurons synthesize serotonin as a neurotransmitter, regulating locomotion and coordinating posture and movement rhythms (Ballion et al., 2002; Takakusaki et al., 2004). Loy et al. (2021) demonstrated that deletion of Sema7A in mice led to a significant increase in serotonergic neuron density across all layers of the adult spinal cord. In SCI models, Sema7A was found to be crucial for the proper restoration of gait and locomotion, as Sema7A-deficient mice exhibited worse locomotor performance. This suggests that Sema7A may also influence serotonergic tract remodeling in adult SCI.

## Conclusion

In this review, NGPs related to SCI are discussed, highlighting their potential as therapeutic targets (**Table 1**). However, while their possible functions have been outlined, the precise molecular mechanisms underlying each NGP’s role in neuronal regeneration remain unclear. Additionally, the interactions between different NGPs have yet to be fully elucidated. Further research is necessary to bridge these knowledge gaps.

**Table 1:** The expression variation and effects of NGPs on neurons and microenvironment, and function recovery in SCI

NGP	Expression variations after SCI	Effects on neurons and microenvironment	Effects on motor function recovery
Netrin-1 in spinal cord tissue	Decrease in the first week, and recovers to equivalent level in the following weeks	Inhibit motor axonal outgrowth and axon regeneration by binding to receptor UNC5; Promote the growth of axons and dendrites on oligodendrocytes	
UNC5	Decrease dramatically after SCI and recovers to the normal level in 40 d		
DCC	Decrease dramatically after SCI and never restores even in 7 mon		
Netrin-1 In serum	Reduce in SCI patients	Inhibit the inflammatory reaction and apoptosis of injured neurons; Restore the autophagic flux in injured neurons	Improve motor function recovery
Slit2	Decline in injured spinal cord	Facilitate the new synapse formation	
Robo1	Increase in injured spinal cord	Inhibit the synapse formation	
EphA7	Increase significantly in spinal cord tissue	Inhibit acute neuron apoptosis	Accelerate recovery of hind limb movement
ephrinA1	Increase in spinal cord tissue		Improve motor performance
EphA3	Increase from 2 dpi to 28 dpi	Inhibit axonal regeneration	
EphA4	+/–	+/–	+/–
ephrinB1	Increase in spinal cord tissue		
EphB1	Increase in spinal cord tissue		
ephrinB2 and EphB2	Start to rise on 1 dpi and peaks by 14 dpi	Activate astrocytes and fibroblasts; Promote formation of astrocytic-meningeal fibroblast scar; Elevate levels of caspase-3 and calpain-1 in neurons	Facilitate spinal injury pain processing
ephrinB1 and EphB1		Elevate levels of caspase-3 and calpain-1 in neurons	Facilitate spinal injury pain processing
EphB3	Increase in astrocytes and motor neurons on 7 dpi	Suppress axonal regeneration	
Sema3A	Be secreted by neurons and fibroblasts in scars around the lesion	Inhibit motor and sensory neuron regeneration	Suppress the recovery of motor and sensory function
Sema3C	Be expressed by macrophages in lesion after SCI and peaks on 7 dpi	Promote microglia polarization, inflammatory cytokine release and subsequently neuronal and myelin damage	
Sema4D	Be highly expressed by motoneurons, endothelial cells and oligodendrocytes in SCI	Activate microglia in the acute phase of SCI; Promote angiogenesis, inflammatory cytokine release; Inhibit neurons regeneration (+/–)	Enhance locomotor recovery after SCI
Sema7A	Be phagocytosed by macrophages in the lesion center, and expressed by reactive astrocytes	Be involved in glial scar formation; Regulate serotonergic neuron density in spinal cord	Promote restoration of gait and locomotion

Inhibitory functions on neuronal regeneration are shown in blue and promotive functions are shown in red. dpi: Days post-injury; NGP: neuronal guidance protein; SCI: spinal cord injury.

SCI is a devastating neurological condition that often leads to severe disability and mortality, placing a significant burden on patients and healthcare systems. Neuronal regeneration and functional recovery following adult SCI are highly limited due to mechanical obstruction caused by scars and the presence of inhibitory local factors. Various NGPs have been shown to play crucial roles in regulating inflammatory responses, neuronal apoptosis, and axonal regeneration and outgrowth in SCI. Exploring the combined local or systemic application of multiple NGPs represents a promising avenue for SCI treatment, warranting further investigation.

## Data Availability

*Not applicable*.
